# Altered Expression of Long Noncoding and Messenger RNAs in Diabetic Nephropathy following Treatment with Rosiglitazone

**DOI:** 10.1155/2020/1360843

**Published:** 2020-01-12

**Authors:** Liwen Zhang, Ying Zhou, Fangfang Zhou, Xialian Yu, Jian Liu, Yunzi Liu, Yufei Zhu, Weiming Wang, Nan Chen

**Affiliations:** ^1^Department of Nephrology, Ruijin Hospital, Shanghai Jiao Tong University School of Medicine, Shanghai 200025, China; ^2^Institute of Nephrology, Shanghai Jiao Tong University School of Medicine, Shanghai 200025, China; ^3^The Key Laboratory of Stem Cell Biology, Shanghai Institutes for Biological Sciences, Chinese Academy of Sciences, Shanghai 200031, China

## Abstract

Diabetic nephropathy (DN) is characterized by metabolic disorder and inflammation. However, the regulatory effects that long noncoding RNAs (lncRNAs) have on the pathogenesis of DN and on the efficacy of rosiglitazone treatment have yet to be clearly defined. Herein, we performed unbiased RNA sequencing to characterize the transcriptomic profiles in db/db diabetic mouse model with or without rosiglitazone treatment that served to improve the phenotypes of DN. Moreover, RNA-seq profiling revealed that the development of DN caused an upregulation in the expression of 1176 mRNAs and a downregulation in the expression of 1010 mRNAs compared to controls, with the expression of 251 mRNAs being returned to normal following treatment with rosiglitazone. Further, 88 upregulated and 68 downregulated lncRNAs were identified in db/db mice compared to controls, 10 of which had their normal expression restored following treatment with rosiglitazone. Bioinformatic analysis revealed that the primary pathways involved in the pathogenesis of DN, and subsequently in the therapeutic effects of PPAR*γ*, are related to inflammatory and metabolic processes. From bioinformatics analysis, lncRNA-AI838599 emerged as a novel molecular mechanism for rosiglitazone treatment in DN through TNF*α*-NF*κ*b pathway. These findings may indicate a new molecular regulatory approach for the development of DN therapeutic agents.

## 1. Introduction

Diabetic nephropathy (DN) is a leading cause of end-stage renal disease (ESRD) in developed nations and is increasing in prevalence in developing countries [1, 2]. The primary clinical features of DN consist of persistent proteinuria, decreased glomerular filtration rate (GFR), and elevated arterial blood pressure [3]. Multiple mechanisms have been proposed to mediate DN, including regulation of metabolic abnormalities that lead to the production of advanced glycation products (AGEs) and reactive oxygen species (ROS), regulating the activation of protein kinase C (PKC), transforming growth factor-beta (TGF-*β*) and the associated inflammatory pathways, as well as limiting the effects of hemodynamic changes [4]. However, inhibition of these pathways has demonstrated only modest effects on clinical manifestations of DN. Currently, the clinical treatment for DN focuses on conventional control of blood glucose and blood pressure, combined with administration of renin-angiotensin-aldosterone system (RAAS) inhibitors to reduce proteinuria. More recent studies have reported beneficial outcomes following administration of SGLT2 inhibitors in improving renal and cardiovascular outcomes in DN patients [5]. However, no specific interventions currently exist that effectively prevent the occurrence and progression of DN. Therefore, it is necessary to explore the pathogenesis and therapeutic targets of DN.

The remarkable ability of thiazolidinediones (TZDs), which are exogenous agonists of peroxisome proliferator-activated receptor gamma (PPAR*γ*), to reduce proteinuria and improve renal pathological features, makes them a promising therapeutic drug for DN. However, the adverse systemic side effects associated with this drug, including fluid retention, cardiovascular complications, hepatotoxicity, and bone fractures [6–8], greatly limit its clinical application. Nevertheless, we cannot deny the value of PPAR*γ* agonists in the protection of kidneys from diabetic injury. PPAR*γ* is expressed in all glomerular and tubular cells [9, 10]. In addition to a well-defined antihyperglycemic role for PPAR*γ* agonists, our previous studies also described a protective role in diabetic and nondiabetic kidney injury for various renal cells, the mechanism of which was independent of the hyperglycemic regulation, and instead functioned primarily through regulation of inflammatory and metabolic-related pathways [11, 12]. However, the specific molecules regulated by PPAR*γ* during inflammatory and metabolic processes remain unclear.

Long noncoding RNAs (lncRNAs) are RNA molecules that are made up of more than 200 nucleotides yet do not encode proteins. Currently, lncRNAs are primarily described as signal molecules since they interact with cellular components, including DNA, RNA, proteins, and chromatin remodeling complexes, to regulate gene expression [13]. Furthermore, animal studies have shown associations between lncRNAs and the development of kidney disease, with increasing evidence emerging suggesting that lncRNAs have an important function in the pathogenesis of DN [14]. Moreover, the expression of lncRNAs is regulated by growth and differentiation and has been shown to exhibit strong tissue and cell specificity [15]. It remains unclear, however, how lncRNAs are involved in the renoprotective role of PPAR*γ* agonists in DN, and whether this mechanism may serve as a potential target in the development of directed therapy for DN.

Given the specific protective effect of PPAR*γ* agonists on kidney tissue and the cell-specific expression of lncRNAs, we hypothesized that lncRNAs may mediate the renal effects of PPAR*γ* agonists and employed PPAR*γ* agonist, rosiglitazone, to intervene db/db diabetic mice and analyzed the lncRNA expression profile alternation in kidney. And the aim of this study is to elucidate the novel renoprotective molecular mechanism of PPAR*γ* agonists and to propose lncRNA targets for diabetic nephropathy treatment, which may provide a new approach to avoid systemic side effects.

## 2. Materials and Methods

### 2.1. Animals

Six-week-old, male C57BL/KsJ db/db diabetic (BKS. Cg-Dock7m+/+Leprdb/Nju; Nanjing Biomedical Research Institute of Nanjing University, China) and nondiabetic littermate control db/m mice were housed in a pathogen-free cage at a constant temperature of 22 ± 2°C and humidity of 50 ± 5%, with normal air CO_2_ levels, 12 h light/dark cycles and *ad libitum* access to standard diet and water. Db/db mice were randomly divided into two groups (dbR and dbdb) and treated with 20 mg/kg/day of rosiglitazone (Sigma-Aldrich; Merck KGaA, Darmstadt, Germany; dbR group) or with a vehicle by gavage for 8 weeks, respectively (from 7 to 15 weeks of age). Animal maintenance and experimental procedures were approved by the Animal Care Committee at the Ruijin Hospital, Shanghai Jiao Tong University School of Medicine (Shanghai, China).

### 2.2. Glucose Tolerance Test and Biochemical Analysis of Urine Samples

After 8 weeks of rosiglitazone or vehicle treatment, urine was collected in metabolic cages to measure the urinary albumin creatinine ratio (ACR). The concentration of albumin and creatinine was detected using the Albuwell M (cat. no. 1011, Exocell, USA) and the Creatinine Companion (cat. no. 1012, Exocell) enzyme-linked immunosorbent assay (ELISA) kits according to the manufacturer's instructions. Mice were fasted for 16 h, before the collection of blood from the caudal vein. Blood glucose concentrations were detected using OneTouch Ultra Glucose Test Strips and an OneTouch UltraEasy Glucometer (Johnson & Johnson, USA) after 0 min, 15 min, 30 min, 45 min, 60 min, 90 min, and 120 min of a 1.5 g/kg intraperitoneal glucose injection.

### 2.3. Kidney Histopathology

Kidneys were removed from 0.3% sodium pentobarbital solution anesthetized mice and immediately fixed in 4% paraformaldehyde at 4°C for 24 h, then embedded in paraffin, and sectioned at 4 *μ*m. Sections were treated with periodic acid-Schiff (PAS) (cat. no. G1008, Servicebio, Wuhan, China) and Masson's trichrome stains (cat. no. G1006, Servicebio) at room temperature according to the manufacturer's protocol. The general histological changes in glomerular and tubular structures were evaluated under the light microscope (DM1000, Leica, Germany).

### 2.4. Transmission Electron Microscopy

Renal cortex tissues were fixed in 2% glutaraldehyde in phosphate-buffered solution (PBS) buffer (pH 7.4). Samples were further incubated with 2% osmium tetroxide in PBS (pH 7.4) for 2 h at 4°C. Ultrathin sections were stained with lead citrate and uranyl acetate and were viewed on a HT770 transmission electron microscope (TEM; Hitachi, Japan) at an accelerating voltage of 80 kV. TEM micrographs were observed for the estimation of globular basement membrane (GBM) thickening and podocyte process fusion.

### 2.5. Western Blot Analysis

Renal cortex tissues were ground and lysed in radioimmunoprecipitation assay (RIPA) buffer containing protease inhibitor cocktail (Bimake, Houston, TX, USA). A total of 30 *μ*g protein of each sample was loaded on sodium dodecyl sulfate (SDS) polyacrylamide gel and transferred to polyvinylidene difluoride (PVDF) membranes (EMD Millipore, Billerica, MA, USA). Membranes were then probed with the following primary antibodies: mouse anti-fibronectin (FN; cat. no. sc-8422; 1 : 300; Santa Cruz Biotechnology, Inc., Dallas, TX, USA), rabbit anti-nephrin (cat. no. ab58968; 1 : 300; Abcam, Cambridge, MA, USA), mouse anti-E-cadherin (cat. no. ab76055; 1 : 1 000; Abcam), rabbit anti-phospho-AMPK (pAMPK; cat. no. 2535; 1 : 1 000; Cell Signaling Technology, Inc., Danvers, MA, USA), mouse anti-vimentin (cat. no. ab8978; 1 : 1 000; Abcam), and mouse anti-*β*-actin antibody (loading control; cat. no. ab8226; 1 : 2 000; Abcam). Subsequently, membranes were incubated with anti-mouse immunoglobulin (Ig)G, horseradish peroxidase- (HRP-) conjugated antibody (cat. no. 7076; 1 : 2 000; Cell Signaling Technology, Inc.) or anti-rabbit IgG, HRP-conjugated antibody (cat. no. 7074; 1 : 2 000; Cell Signaling Technology, Inc.) for 1 h at room temperature on a horizontal shaker.

Membranes were visualization using Immobilon Western Chemiluminescent HRP Substrate (cat. no. WBKLS0100; EMD Millipore) and the Luminescent Imaging Workstation (Tanon, Shanghai, China), and band intensities were quantified using ImageJ (version number 1.8.0; National Institutes of Health, Bethesda, MD, USA).

### 2.6. RNA Isolation and RNA Sequencing Analysis

Total RNA was extracted from the renal cortex of each mouse (*n* = 3 for each group) using TRIzol reagent (Invitrogen, Carlsbad, CA, USA). The integrity and purity of the RNA were determined via the Agilent 2100 Bioanalyzer system (Agilent, Santa Clara, CA, USA). All samples with RIN < 8 and 28S/18S < 1 were excluded from the analysis. Ribosomal RNA was removed with an Epicentre Ribo-zero™ rRNA removal kit (Epicentre, USA) according to the manufacturer's instructions. Sequencing libraries were generated using the rRNA-depleted RNA by NEBNext® Ultra™ Directional RNA Library prep kit from Illumina® (NEB, USA) and sequenced using an Illumina HiSeq X Ten platform from Novogene (Beijing, China), which generated 150 bp paired reads.

### 2.7. Analysis of RNA Sequencing Data

FASTQ raw reads were firstly processed through in-house Perl scripts to obtain clean reads, which were aligned with the Ensembl reference mouse transcriptome (GRCm38.p2) using TopHat v2.0.9 [16]. The resulting mapped reads for each sample were assembled using Scripture Beta2 [17] and Cufflinks (v2.1.1) [18] in a reference-based approach. Cufflinks were used to calculate fragments per kilobase of exon per million mapped reads (FPKMs) for the lncRNAs and coding genes identified in each sample that mapped to a specific fragment. Gene FPKMs were computed by adding the transcript FPKMs in each gene group. Cufflinks provide statistical computation based on negative binomial distribution, allowing for the identification of differentially expressed digital transcripts or genes. Transcripts with an adjusted *P* value (*q* value) <0.05 were considered to be differentially expressed.

### 2.8. Target Gene Prediction of lncRNAs and Enrichment Analysis

To identify lncRNAs and mRNAs at the same loci, we searched coding genes 100 kb upstream and downstream of the lncRNA, while for lncRNA coexpressed with mRNAs, we identified correlations between lncRNA expression with those of coding genes using the Pearson correlation coefficient method with the absolute value of the threshold set to 0.95 when searching common expression modules. We then analyzed the function of target genes via functional enrichment analysis. Specifically, Gene Ontology (GO; http://www.geneontology.org) enrichment analysis of differentially expressed genes (DEGs) or lncRNA target genes was performed using the goseq R package (Bioconductor), during which gene length bias was corrected. GO results with corrected *P* values (*q* values) <0.05 were considered significantly enriched by DEGs. We also employed KOBAS (2.0) [19] software to assess the statistical enrichment of DEGs or lncRNA target genes in Kyoto Encyclopedia of Genes and Genomes (KEGG; http://www.genome.jp/kegg/) pathways. A pathway with *q* < 0.05 was defined as significantly enriched in DEGs compared to the whole genomic background.

### 2.9. Gene Set Enrichment Analysis

Gene set enrichment analysis (GSEA) was also performed using GSEA v3.0 software [20] for the complete mRNA expression dataset. The Molecular Signatures Database (MSigDB) (http://software.broadinstitute.org/gsea/msigdb) was used to provide predefined gene sets.

### 2.10. RNA Sequencing qRT-PCR Validation

Total RNA isolated from the renal cortices of mice (*n* = 5 for each group) were processed by reverse transcription to create cDNA using a high capacity cDNA reverse transcription kit (Applied Biosystems; Thermo Fisher Scientific, Inc., Waltham, MA, USA) according to the manufacturer's instructions. Real-time quantitative reverse transcription polymerase chain reaction (qRT-PCR) was performed using SYBR® Premix Ex Taq™ (TAKARA, Japan) and the StepOnePlus real-time PCR system (Applied Biosystems) with primer sequences listed in Supplementary [Supplementary-material supplementary-material-1]. Expression levels of each lncRNA were calculated after normalizing with *β*-actin. The results were analyzed using the comparative cycle threshold (2^−∆∆Ct^) method.

### 2.11. Statistical Analyses of Phenotypic Data

Data for each study group are expressed as mean ± standard error of the mean (SEM). Comparisons between two groups were performed using unpaired Student's *t*-tests after determination of data distributions and variance. One-way analysis of variance (ANOVA) followed by Tukey multiple comparisons test was performed when more than two groups were involved in the analysis. All tests were two-tailed, with a *P* < 0.05 considered to be a statistically significant result.

## 3. Results

### 3.1. Rosiglitazone Protected Db/Db Mice against Kidney Injury

**Figure 1:**
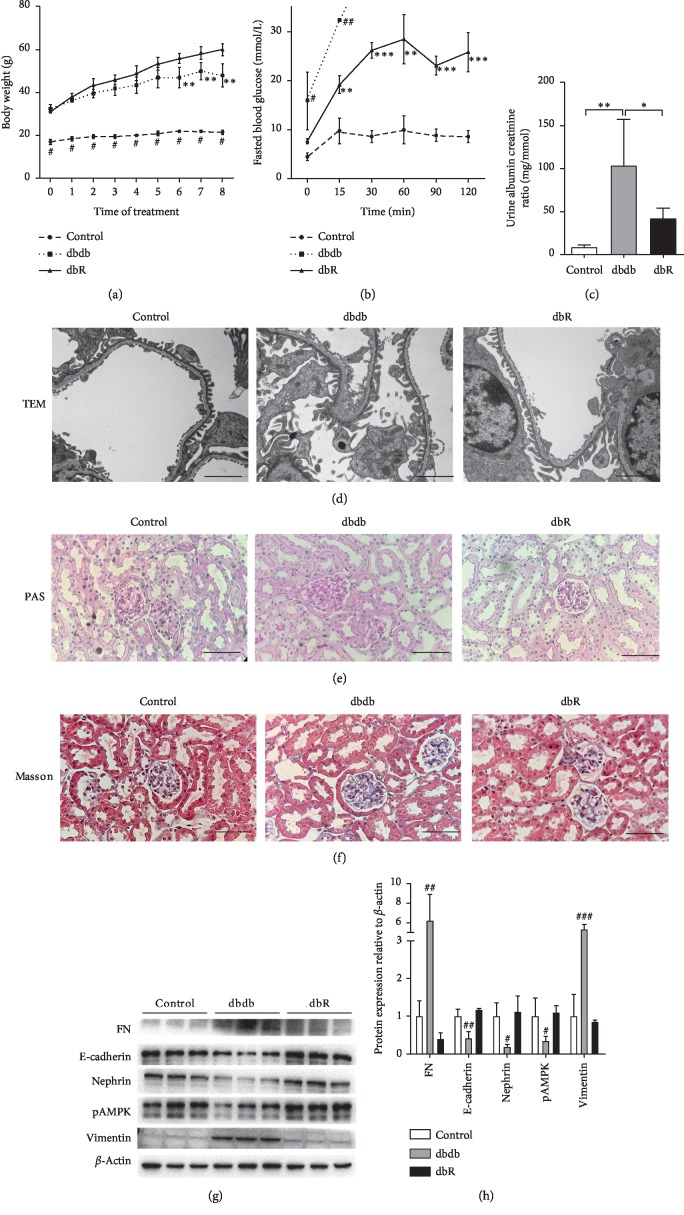
Rosiglitazone treatment protected against diabetic nephropathy in db/db mice. (a) Body weight levels were evaluated weekly in the control, dbdb, and dbR groups. Data are presented as the mean ± SEM (*n* = 6 per group). ^#^*P* < 0.001 compared to the other groups at the same time point, ^*∗∗*^*P* < 0.01 compared to dbR at the same time point. (b) Intraperitoneal glucose tolerance test (IPGTT) in rosiglitazone-treated and untreated control and db/db mice. After eight weeks of treatment with rosiglitazone, mice were fasted for 16 hours and injected with glucose (1.5 g/kg I.P). Blood glucose levels were measured at 0 min, 15 min, 30 min, 60 min, 90 min, and 120 min after injection. The data omitted in the dbdb group are due to blood glucose levels above the upper limit of instrument detection. ^*∗∗*^*P* < 0.01 compared to the control group; ^*∗∗∗*^*P* < 0.001 compared to the control group; ^#^*P* < 0.05 compared to the other two groups; ^##^*P* < 0.01 compared to the other two groups. Data are represented as mean ± SEM. *n* = 6 per group. (c) After 8 weeks of treatment, urinary albumin creatine ration (ACR) in control, dbdb, and dbR mice was determined. *n* = 6 per group. ^*∗*^*P* < 0.05, ^*∗∗*^*P* < 0.01, as indicated. Representative photomicrographs depicting (d) transmission electron microscope (TEM), (e) periodic acid-Schiff (PAS) staining, and (f) Masson's trichome staining in the control, dbdb, and dbR groups after the 8-week experimental period. Scale bars: (d) 2 *μ*m and (e, f) 50 *μ*m. (f) Western blot analysis of fibronectin (FN), E-cadherin, nephrin, pAMPK, vimentin, and *β*-actin expression in the renal cortices of control, dbdb and dbR mice. (g) Densitometric analysis of western blot results. (h) Relative band intensity was normalized to the *β*-actin signal. Data are presented as the mean ± SEM (*n* = 6 per group). ^#^*P* < 0.05, ^##^*P* < 0.01, ^###^*P* < 0.001, as compared to the other groups.

To determine whether rosiglitazone served to protect kidneys from damage during DN, we firstly evaluated the renal phenotypes of db/m and db/db mice with and without rosiglitazone treatment. During the 8 weeks of administration, the body weights of db/db mice (both dbdb and dbR groups) were significantly higher than those of db/m (control) mice of the same age. Further, beginning at the 6^th^ week of treatment, the body weight of the dbR group was found to be significantly higher than that of the dbdb group (Figure 1(a)). After 8 weeks of administration (15 weeks of age), the results from the intraperitoneal glucose tolerance test (IPGTT) suggested that rosiglitazone significantly improved glucose tolerance in db/db mice (Figure 1(b)). Moreover, the urinary ACR was determined to be significantly higher in the dbdb group compared to the control group, while rosiglitazone treatment (dbR group) effectively reduced the urinary ACR of db/db mice (Figure 1(c)). Additionally, transmission electron microscopy (TEM) micrographs showed apparent podocyte foot process fusion in the dbdb group compared with the control group, and recovery was observed in the dbR group (Figure 1(d)). These results indicated that treatment with rosiglitazone protects against the development of podocyte lesions in DN and reduces proteinuria.

Moreover, PAS staining revealed obvious glomerular hypertrophy and substantial mesangial matrix accumulation in the glomeruli of the dbdb group compared to the control group (Figure 1(e)). Furthermore, Masson's trichome staining revealed apparent mesangial area expansion, glomerulosclerosis, and tubulointerstitial fibrosis in the dbdb group (Figure 1(f)). Treatment with rosiglitazone resulted in only minor differences compared with the control group, without apparent development of pathological characteristics in the light micrographs.

Western blot analysis revealed that, compared with the control group, the expression of E-cadherin, nephrin, and pAMPK in the kidney cortex tissue was significantly downregulated in the dbdb group, while the expression of FN and vimentin was increased (Figures 1(g) and 1(h)). However, treatment with rosiglitazone served to reverse the altered expression of these proteins (Figures 1(g) and 1(h)). These data suggest that rosiglitazone improves podocyte injury, glomerulosclerosis, and epithelial-mesenchymal transdifferentiation (EMT) in DN.

### 3.2. Rosiglitazone Treatment Reversed Partial Differentially Expressed mRNAs and lncRNAs in Diabetic Nephropathy

**Figure 2:**
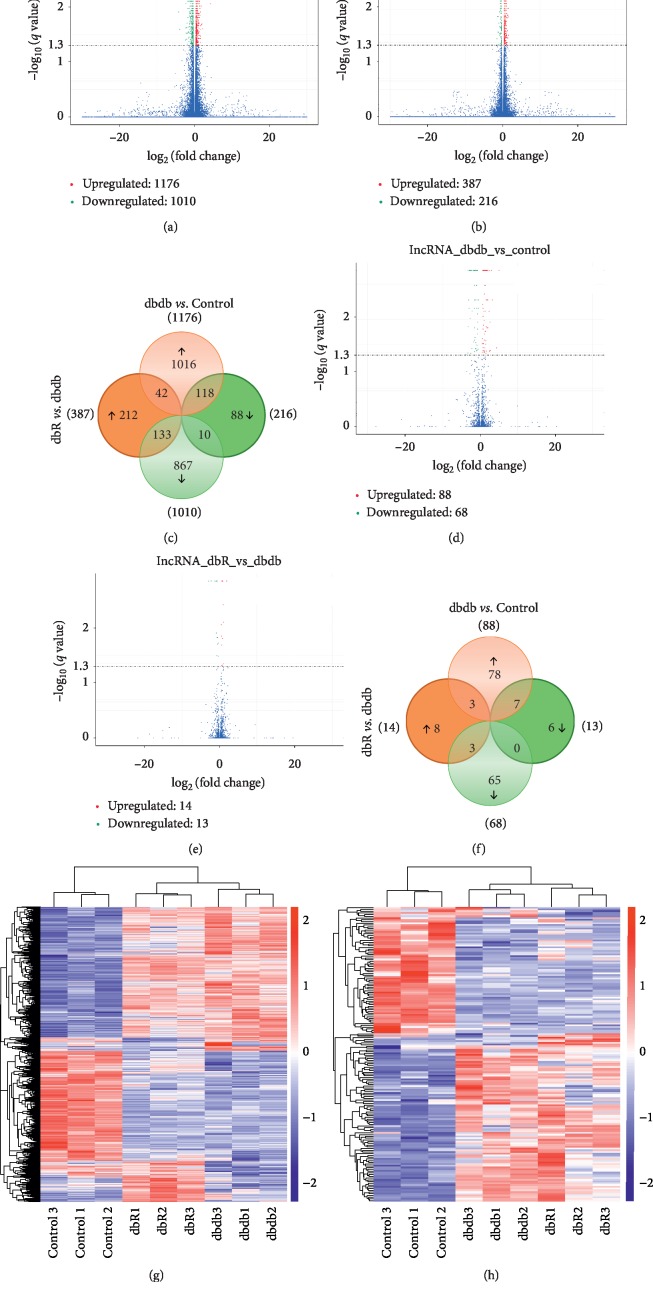
Differentially expressed mRNAs and lncRNAs in the renal cortices of diabetic nephropathic mice. Volcano plot for comparison of (a) mRNA expression between the dbdb and control group, (b) mRNA expression between the dbR and dbdb group, (d) lncRNA expression between the dbdb and control group, and (e) lncRNA expression between the dbR and dbdb group. On the right side, the red color is indicative of the upregulated genes (*q* < 0.05); on the left side, the green color is indicative of the downregulated gene (*q* < 0.05). The blue points indicate mRNAs that were not statistically significant (*q* > 0.05). Venn diagrams illustrate the number of significantly differentially expressed (c) mRNAs and (f) lncRNAs. The overlapping areas represent the coregulated genes in both dbdb/control and dbR/dbdb analyses. Differentially expressed (g) mRNAs and (h) lncRNAs (*q* < 0.05) in dbdb/control or dbR/dbdb analyses were assessed using hierarchical clustering. Each row represents a single gene expression, and each column represents one tissue sample.

To identify the molecular mechanisms responsible for the renal protective effects elicited by rosiglitazone, we extracted total RNA from the renal cortices of 3 mice in each of the control, dbdb, and dbR groups and performed RNA-seq analysis. All RNA-seq data have been deposited in the NCBI's Gene Expression Omnibus (accession no. GSE139987). The bioinformatic analysis consisted of two independent comparisons: db/db vs. normal control (dbdb vs. control) and db/db mice treated with rosiglitazone (dbR) vs. mice treated with a vehicle (dbdb). Volcano plots provided an overview of the differential expression of mRNAs (Figures 2(a)–2(c)) and lncRNAs (Figures 2(d)–2(f)). A threshold of *q* < 0.05 was the setpoint to confirm differential expression. Results indicate that 1176 mRNAs were differentially upregulated (Supplementary [Supplementary-material supplementary-material-1]) and 1010 were downregulated (Supplementary [Supplementary-material supplementary-material-1]) in the dbdb mice compared to the control group. A further 378 mRNAs were determined to be differentially upregulated (Supplementary [Supplementary-material supplementary-material-1]), and 216 were downregulated (Supplementary [Supplementary-material supplementary-material-1]) in the dbR mice compared to the dbdb group. There were also 118 mRNAs identified as upregulated in dbdb compared to the control yet downregulated in the dbR mice compared to those in the dbdb group (Figure 2(c), Supplementary [Supplementary-material supplementary-material-1]). Alternatively, 133 mRNAs were differentially downregulated in dbdb compared to the control yet were upregulated in the dbR mice compared to the dbdb group (Figure 2(c), Supplementary [Supplementary-material supplementary-material-1]). Moreover, 88 lncRNAs were identified as upregulated (Supplementary [Supplementary-material supplementary-material-1]) and 68 were downregulated (Supplementary [Supplementary-material supplementary-material-1]) in dbdb mice compared to the control, while 14 were upregulated (Supplementary [Supplementary-material supplementary-material-1]) and 13 were downregulated (Supplementary [Supplementary-material supplementary-material-1]) in dbR mice compared to the dbdb group. Further, 7 lncRNAs were upregulated in dbdb compared to the control yet downregulated in dbR mice compared to dbdb (Figure 2(f), Supplementary [Supplementary-material supplementary-material-1]), and another 3 lncRNAs were downregulated in dbdb compared to control yet upregulated in dbR compared to dbdb (Figure 2(f), Supplementary [Supplementary-material supplementary-material-1]). Heatmaps were constructed to indicate the shared mRNA and lncRNA expression between these three study groups (Figures 2(g)and 2(h)).

### 3.3. The Differentially Expressed mRNAs and lncRNA-Targeted mRNAs Were Associated with Specific GO Functions and KEGG Pathways

**Figure 3:**
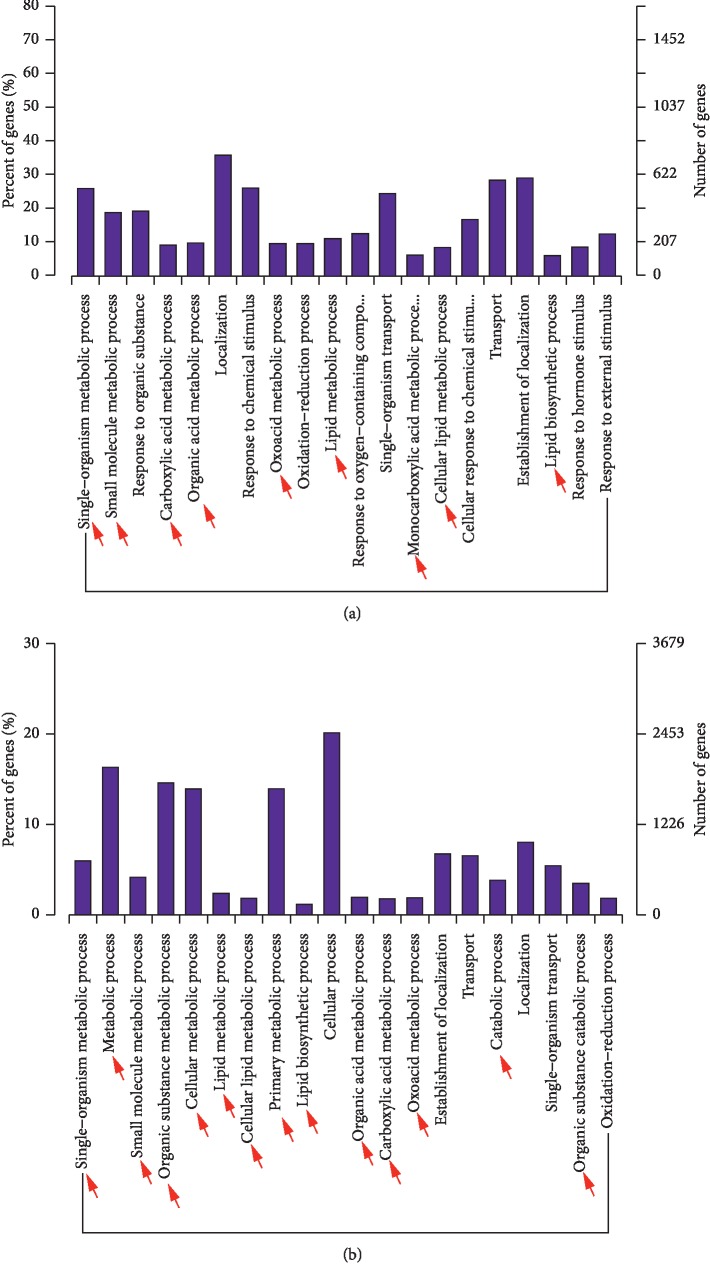
GO analysis of differentially expressed mRNAs. GO term enrichment analysis of regulated genes as they relate to biological processes for (a) dbdb compared to the control mice and (b) dbR compared to the dbdb mice. GO, Gene Ontology; BP, biological process.

**Figure 4:**
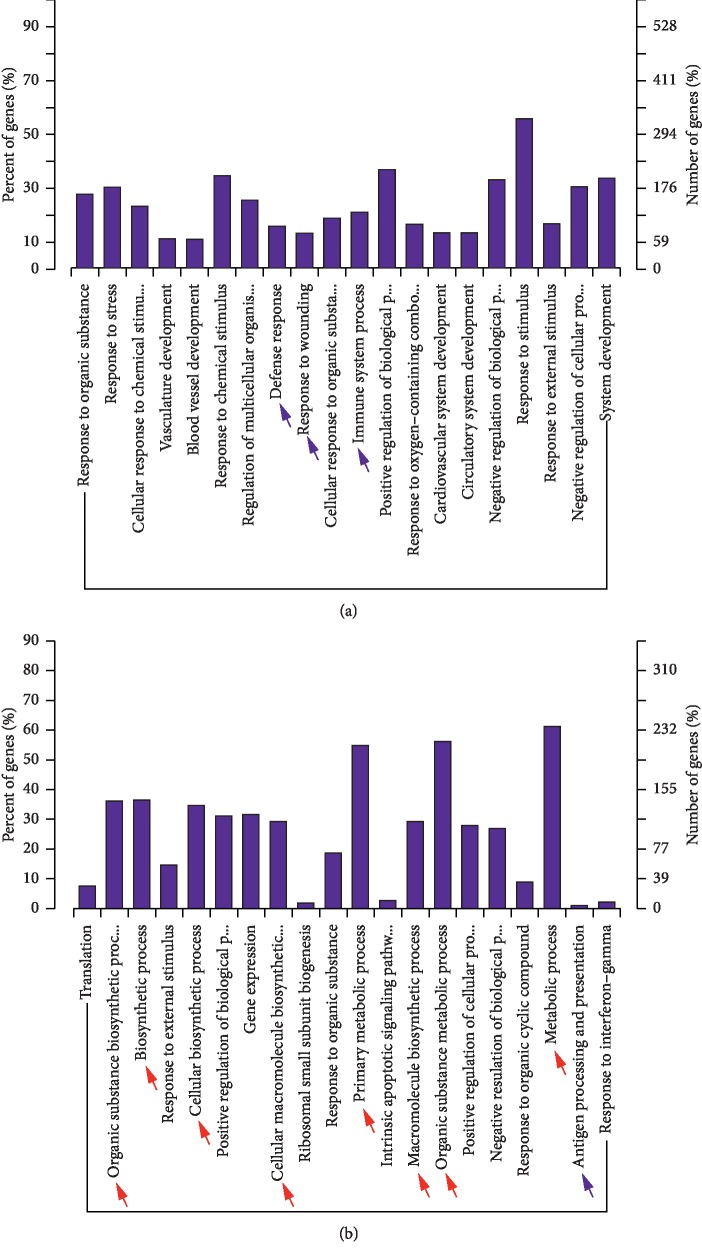
GO analysis of differentially expressed lncRNAs coexpressed with mRNAs. GO term enrichment analysis of regulated lncRNAs coexpressed with mRNAs as they relate to biological processes for (a) dbdb compared to the control mice and (b) dbR compared to the dbdb mice. GO, Gene Ontology; BP, biological process.

The number of differentially expressed lncRNAs colocated target mRNAs (the mRNAs are listed in Supplementary [Supplementary-material supplementary-material-1]) was too few to perform GO analysis. However, the histogram depicting the GO enrichment of differentially expressed mRNAs (Figure 3(a), Supplementary [Supplementary-material supplementary-material-1]) and differentially expressed lncRNAs coexpressed mRNAs (the mRNAs are listed in Supplementary [Supplementary-material supplementary-material-1]) (Figure 3(b), Supplementary [Supplementary-material supplementary-material-1]) in dbdb mice compared to the control group reveals that a large proportion of these mRNAs and lncRNAs are associated with metabolic functions. Alternatively, the analysis comparing dbR and dbdb mice contained a high proportion of differentially expressed mRNAs associated with inflammatory functions (Figure 4(a), blue arrow; Supplementary [Supplementary-material supplementary-material-1]), while the differentially expressed lncRNAs that were coexpressed with mRNAs were found to be more enriched in metabolic-related function (Figure 4(b), red arrow; Supplementary [Supplementary-material supplementary-material-1]).

**Figure 5:**
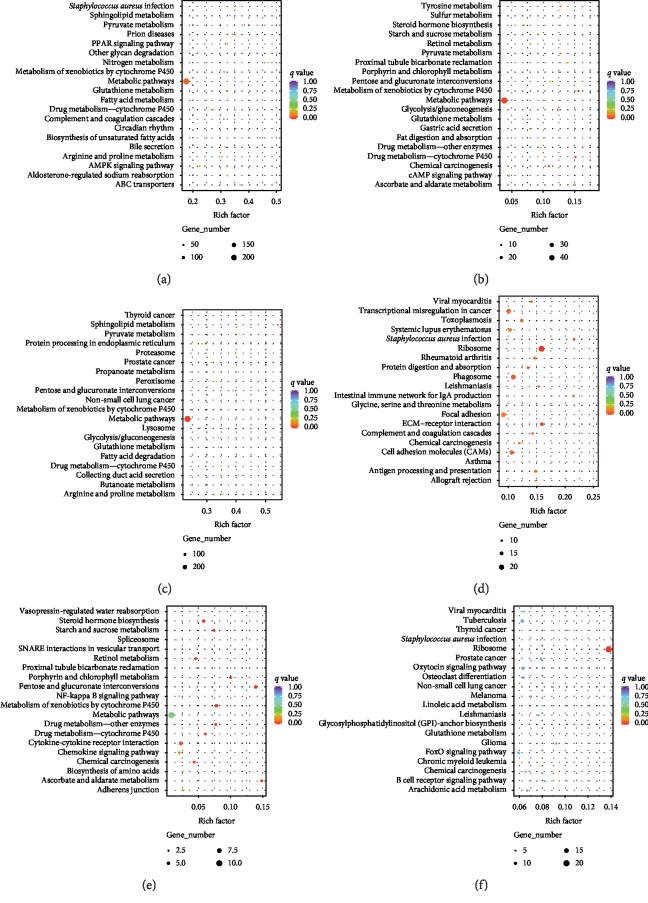
KEGG enrichment scatter plot of differentially expressed genes. The top 20 significantly enriched KEGG pathways associated with differentially expressed mRNAs for (a) dbdb compared to the control group and (d) dbR compared to the dbdb group. The top 20 significantly enriched KEGG pathways associated with differentially expressed lncRNAs colocated with mRNAs for (b) dbdb compared to the control group and (e) dbR compared to the dbdb group. The top 20 significantly enriched KEGG pathways associated with differentially expressed lncRNAs coexpressed with mRNAs for (c) dbdb compared to the control group and (f) dbR compared to the dbdb group. Notes: rich factor: input number/background number. Input number: number of corresponding genes associated with differential mRNAs enriched in the KEGG pathway and background number: number of genes in the pathway.

The top 20 significantly enriched pathways are presented in Figure 5, and the degree of KEGG enrichment is expressed as rich factor, *q* value, and the number of genes enriched in each pathway. In the analysis between db/db and control mice, the differentially expressed mRNAs (Figure 5(a), Supplementary [Supplementary-material supplementary-material-1]) and differentially expressed lncRNAs colocated (Figure 5(b), Supplementary [Supplementary-material supplementary-material-1]) or coexpressed (Figure 5(c), Supplementary [Supplementary-material supplementary-material-1]) with mRNAs were most strongly associated with metabolic pathways as observed by the high enriched gene numbers and low *q* values. However, differentially expressed mRNAs were also found to be highly associated with inflammatory processes (*q* value =0.053), including *Staphylococcus aureus* infection as well as complement and coagulation cascades (Figure 5(a)). More specifically, within the comparison of dbR and dbdb mice, both the differentially expressed mRNAs (Figure 5(d), Supplementary [Supplementary-material supplementary-material-1]) and the differentially expressed lncRNAs coexpressed with mRNAs (Figure 5(f), Supplementary [Supplementary-material supplementary-material-1]) were most strongly associated with the ribosome pathway, and differential mRNAs were also enriched significantly in inflammatory-related pathways, including *Staphylococcus aureus* infection, intestinal immune network for IgA production, complement and coagulation cascades, and antigen processing and presentation (Figure 5(d), Supplementary [Supplementary-material supplementary-material-1]). Alternatively, the differentially expressed lncRNAs that were colocated with mRNAs were determined to be significantly enriched in metabolic-related pathways, including steroid hormone biosynthesis, starch and sucrose metabolism, pentose and glucuronate interconversions, and metabolism of xenobiotics by cytochrome P450 (Figure 5(e), Supplementary [Supplementary-material supplementary-material-1]); the metabolism pathways and NF*κ*b signaling pathway were also notably associated with these differentially expressed mRNAs and however did not reach statistical significance (Figure 5(e), Supplementary [Supplementary-material supplementary-material-1]). Taken together, it appears that the differentially expressed mRNAs identified in dbdb compared to dbR mice were more enriched in inflammation-related pathways rather than those associated with metabolic processes.

### 3.4. Inflammation-Related Genes Were Significantly Enriched in dbR Compared to Dbdb Mice following GSEA

**Figure 6:**
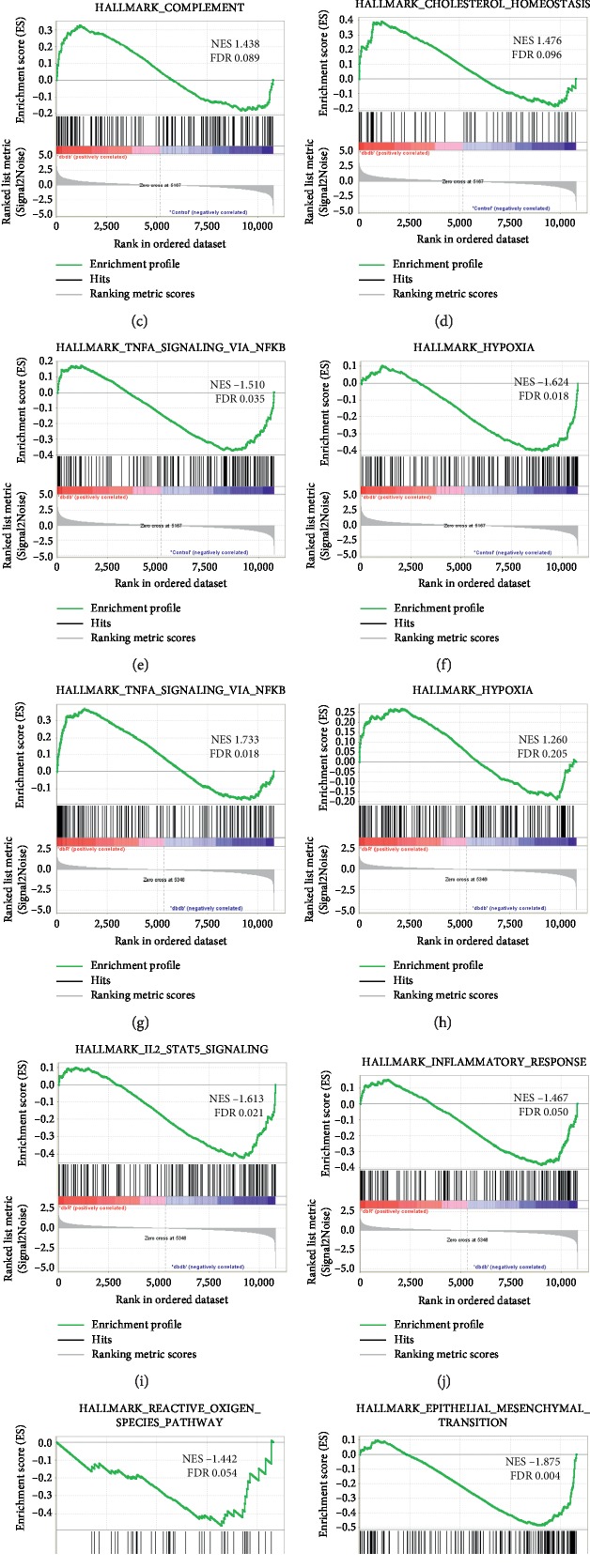
Representative significantly enriched gene sets from GSEA for the global mRNA expression profile. Upregulated gene sets identified in dbdb mice (a–d) and upregulated genes in control mice (e, f) following analysis of dbdb compared to control groups. Upregulated gene sets in dbR mice (g, h), and upregulated genes in dbdb mice (i–l) following analysis of dbR compared to dbdb group. In every thumbnail, the green curve represents the evolution of the density of the genes identified in the RNA-seq. The false discovery rate (FDR) is calculated by comparing the actual data with 1000 Monte Carlo simulations. The NES (normalized enrichment score) computes the density of modified genes in the dataset with the random expectancies, normalized by the number of genes found in a given gene cluster, to account for the size of the cluster.

The complete mRNA expression dataset was submitted to GSEA to extract biologically relevant information. The GSEA of differentially expressed mRNA in dbdb mice compared to the control group revealed enrichment in three inflammation-related (Figures 6(a)–6(c)) and one metabolism-related gene sets (Figure 6(d)) that were significantly upregulated in dbdb mice, and the TNF*α* signaling via NF*κ*b (Figure 6(e)) as well as the hypoxia gene sets (Figure 6(f)) were significantly upregulated in the control group (Supplementary Tables [Supplementary-material supplementary-material-1], [Supplementary-material supplementary-material-1]). Furthermore, within the analysis of dbR mice compared to those in the dbdb group, both the TNF*α* signaling via NF*κ*b (Figure 6(g)) and the hypoxia gene sets (Figure 6(h)) were upregulated in dbR mice, while another two inflammation-related gene sets (Figures 6(i) and 6(j)) and the reactive oxygen species pathway gene set (Figure 6(k)) were upregulated in dbdb mice (Supplementary Tables [Supplementary-material supplementary-material-1], [Supplementary-material supplementary-material-1]). We also noted that the epithelial-mesenchymal transition gene set exhibited the highest NES value with an FDR <25% and was associated with differentially expressed mRNAs in dbR mice compared to the dbdb group (Figure 6(l), Supplementary [Supplementary-material supplementary-material-1]), which may contribute to the renal protective mechanism of rosiglitazone in DN.

### 3.5. Validation of Representative lncRNAs

**Figure 7:**
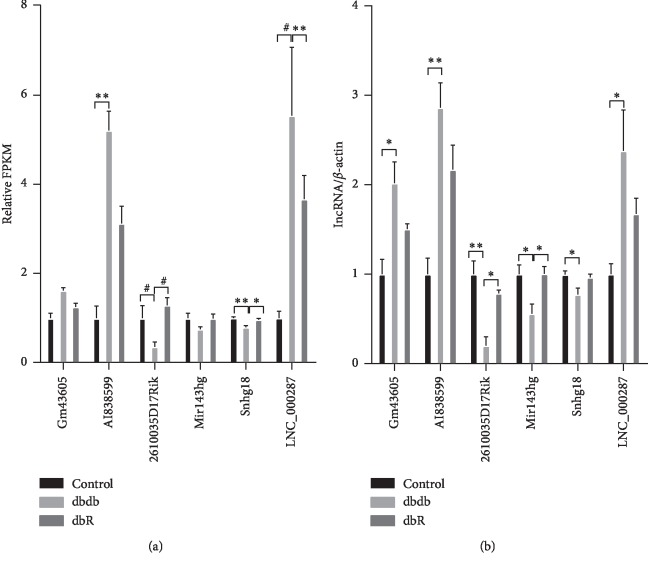
Differential expression of lncRNAs validated by qRT-PCR. (a) The relative expression of lncRNAs as identified via RNA-seq analysis. The fragments per kilobase of exon per million mapped reads (FPKM) values in the control group were normalized to 1, and the relative expressions in the dbdb and dbR groups are shown. ^*∗*^*P* < 0.05, ^*∗∗*^*P* < 0.01, and ^#^*P* < 0.001, as indicated. (b) The relative expressions of lncRNAs in qRT-PCR were validated in the control, dbdb, and dbR mice (*n* = 6 for each group). ^*∗*^*P* < 0.05, ^*∗∗*^*P* < 0.01.

The RNA-seq results were verified via qRT-PCR. Six lncRNAs that exhibited opposite expression patterns in dbdb vs. control and dbR vs. dbdb analyses were randomly chosen (Figure 7(a)) and qualified by qRT-PCR using total RNA from kidney cortices of db/db mice. The results revealed that Gm43605, AI838599, and a novel lncRNA, namely, LNC_000287 (the location and sequence are listed in Supplementary [Supplementary-material supplementary-material-1]) were significantly upregulated in the dbdb group compared to those in the control group, and that they were downregulated, without statistical significance, in the dbR group compared to the dbdb mice (Figure 7(b)). Meanwhile, lncRNA 2610035D17Rik, Mir143 hg, and Snhg18 were identified as significantly downregulated in the renal samples of db/db mice compared to the controls yet upregulated in dbR mice compared to the dbdb group (Figure 7(b)). Thus, the qRT-PCR results were consistent with those of the RNA-seq analysis, which provides further validation that these representative lncRNAs may be related to the pathogenesis and development of DN and the renal protective mechanism exhibited by rosiglitazone.

More importantly, we found that three coexpressed mRNAs of lncRNA-AI838599 (*Gadd45b*, *Per1*, and *Sphk1*) (Supplementary [Supplementary-material supplementary-material-1]) were in the Hallmark TNF*α* signaling via NF*κ*b gene set (MSigDB). All of the three mRNAs were core enrichment, making main contributions to the enrichment score of the gene set, and were upregulated in dbdb compared to the control yet were downregulated in the dbR mice compared to the dbdb group (Table 1). These findings propose lncRNA-AI838599 as a key rosiglitazone-regulated gene in DN treatment.

## 4. Discussion

PPAR*γ* is expressed in all kidney cells, and the renoprotective effect of PPAR*γ* agonists is kidney-specific and independent of cardiovascular and systemic side effects. Thus, it is reasonable to speculate that lncRNA, which is poorly conserved and exhibits strong tissue and cell specificity [21], contributes to the renal protection elicited by PPAR*γ* agonists. We proposed a list of lncRNAs involved in the renoprotection of PPAR*γ* agonists through RNA-seq and predicted their biological functions through bioinformatics analysis.

Our previous studies described a protective role for rosiglitazone, a PPAR*γ* agonist, in mouse models of diabetic and nondiabetic nephropathy [11, 12]. We determined that the protective mechanism employed by this drug involved reducing inflammation, improving metabolism, and protecting against oxidative stress. However, we also noted adverse systemic and cardiovascular side effects following treatment with rosiglitazone (such as the elevated body weight and increased serum total and low-density lipoprotein cholesterol, the latter data not shown), suggesting that kidney-specific mechanisms occur in response to PPAR*γ* agonist treatment. The aim of the current study was to, therefore, elucidate the downstream molecular mechanisms involved in renal protection by PPAR*γ* agonists. Employing the db/db diabetic mouse models, we confirmed that rosiglitazone reduced blood glucose and proteinuria, improved renal pathological damage, and prevented transdifferentiation in mice with DN. We then used RNA-seq analysis and a combination of different bioinformatic analyses to identify downstream coding and long noncoding transcripts as well as molecular pathways that may be involved in the kidney-specific response of PPAR*γ* agonists in DN.

We evaluated the coding and long noncoding gene expression in the renal cortices of control (db/m, control group), diabetic (db/db, dbdb group), and rosiglitazone-treated diabetic (db/db rosiglitazone, dbR group) mice. Differential expression analysis revealed that 303 mRNAs and 13 lncRNAs (approximately 15% of differentially expressed mRNAs and >8% of differentially expressed lncRNAs induced by DN, and nearly 50% of the mRNAs and lncRNAs regulated by rosiglitazone) were coregulated by both diabetes and rosiglitazone in DN, with the expression pattern of approximately 80% of the coregulated mRNAs and lncRNAs reversed by rosiglitazone treatment. Those genes that had their expression restored following treatment with rosiglitazone are likely involved in protection against DN. However, the renal implications and functions of genes that were exacerbated following treatment with rosiglitazone are unclear. For example, in healthy human kidneys, heparin-binding EGF-like growth factor (HBEGF) expression is weak and restricted to tubules and vascular smooth muscle cells [22]. Moreover, Bollée et al. [22] found that expression of HBEGF was induced in crescentic rapidly progressive glomerulonephritis (RPGN) and that the HBEGF-EGFR pathway activation occurred in podocytes resulting in the development of RPGN. However, the cause of the downregulation in HBEGF expression within the kidney of db/db mice compared to the control group and the further downregulation following treatment with rosiglitazone remains unclear and may be related to the systemic side effects of rosiglitazone. Furthermore, it is unclear whether the reversed effects on gene expression elicited by rosiglitazone were the result of indirect glycemic control or downstream PPAR*γ* activation. Hence, further mechanistic studies are needed to elucidate the genes most critical to inducing changes in metabolic and inflammatory processes within the kidney and their relationship with the differentially expressed lncRNAs.

Hinder et al. [23] analyzed the tissue-specific effects of the diabetic drug, pioglitazone, through differential expression analysis and self-organizing map (SOM) analysis of various tissues following treatment with pioglitazone. Our study analyzed the coding and noncoding genes via whole transcriptomics analysis to determine the effects of rosiglitazone in the kidney. Consistent with their reports, our data suggest that changes in tissue remodeling and EMT are ameliorated following treatment with a PPAR*γ* agonist. Moreover, epithelial-mesenchymal transition was the most significantly upregulated gene set identified in the dbdb mice compared to the dbR group in our study. Genes associated with promoting proliferation and fibrosis such as *Grem1*, *Grem2* [24, 25], and *Ctgf* were significantly upregulated in the kidney cortices of diabetic mice; however, the expression of these genes was downregulated following treatment with rosiglitazone. Furthermore, increasing attention has been given to the potential role that Kruppel-like factors (KLFs) have in kidney disease, especially in diabetic nephropathy [26, 27]. Specifically, the profibrotic KLF10 and KLF11 and the proinflammatory KLF13 [28, 29] have been shown to be significantly upregulated in DN, yet, in our study, rosiglitazone treatment served to reverse this overexpression, which may underline the new renoprotective mechanism of rosiglitazone, and further investigations are needed to confirm their role in DN.

In addition, the metabolic reprogramming mechanism previously described for lncRNAs in DN, and following rosiglitazone treatment, is not yet clear. Recent studies have shown that lncRNAs play an important role in kidney diseases including DN. For example, lncRNA-MALAT1, lncRNA-Gm4419, and lncRNA-Tug1 modulate the progression of diabetic nephropathy by regulating inflammation or metabolism [30–32]. In our study, specific lncRNAs were identified in the renal cortex of the db/db mice with or without rosiglitazone treatment, some of which exhibited altered expression patterns in DN. Following treatment with rosiglitazone, the expression of these lncRNAs returned to normal, corresponding with improvement in pathophysiological symptoms. The target genes of the differentially expressed lncRNAs in dbdb compared to dbR mice were found to be primarily associated with metabolic processes, the specific molecular mechanism of which is an important focus of our ongoing research. Further, it remains unclear whether these differentially expressed genes associated with metabolic processes are involved in the development of diabetes or whether they are the result of the disease.

Noticeably, we found that lncRNA-AI838599 might be a novel molecular mechanism for rosiglitazone treatment in DN and provide a new target for DN treatment. We confirmed the upregulated expression of lncRNA-AI838599 in DN through RNA-seq and qPCR, which was reversed by rosiglitazone treatment. However, the reversed expression by rosiglitazone was not statistically significant because of the limitation of the sample size, we think. In addition, the relationship between lncRNA-AI838599 and kidney is also reported previously. GSE87899 (GEO DataSets) [33] showed increased lncRNA-AI838599 expression in the kidney of D2.B6-Ins2 ^Akita^/MatbJ DN mouse model compared to the wild type. The similar expression trend of lncRNA-AI838599 was also seen in miR-25 knockdown mice that show similar phenotypes of DN such as proteinuria, extracellular matrix accumulation, and podocyte foot process effacement [34]. Although the lncRNA expression profile change caused by rosiglitazone administration was generally enriched in metabolic-related pathways, the TNF*α* signaling via NF*κ*b gene set was regulated in the opposite direction by DN and rosiglitazone treatment, and including three lncRNA-AI838599 coexpressed mRNAs (the molecular function of lncRNA predicted by bioinformatics), Gadd45b, Per1, and Sphk1, as core enrichment. Gadd45b, Per1, and Sphk1 are all induced by TNF*α*-NF*κ*b activation in various models [35–37]. Taken together, the TNF*α*-NF*κ*b pathway is a key mechanism for the improvement of DN by rosiglitazone, and bioinformatics analysis predicts that lncRNA-AI838599 regulates the TNF*α*-NF*κ*b pathway by effecting its target genes, *Gadd45b*, *Per1*, and *Sphk1*.

In summary, our RNA-seq analysis of rosiglitazone treatment in DN suggests that a range of coding and noncoding transcripts may be involved in the pathophysiological processes of DN and in the renal protective effects elicited by PPAR*γ* activation. The lncRNA expression profile provides a basis for understanding the renal-specific protective mechanism of PPAR*γ* agonists and may provide a theoretical basis for new therapeutic strategies for diabetic nephropathy. Moreover, we propose lncRNA-AI838599 as a novel therapeutic target for DN treatment, which is our focus of the future research.

## Figures and Tables

**Table 1:** Differential expression and GSEA details of lncRNA-AI838599 coexpressed targeted mRNAs in the TNF*α* signaling via NF*κ*b gene set.

Transcript ID	Gene symbol	Differential expression			GSEA details	
		Log2 (fold change)	*P* value	Q value	Running enrichment score	Core enrichment
*dbdb vs. control*							
ENSMUST00000015456	Gadd45b	1.33966	0.00015	0.00379	−0.1283	Yes
ENSMUST00000021271	Per1	1.58381	0.00005	0.00141	−0.0646	Yes
ENSMUST00000063446	Sphk1	2.60514	0.03165	0.26383	−0.0443	Yes

*dbR vs. dbdb*							
ENSMUST00000015456	Gadd45b	−0.44219	0.0991	0.53172	0.2098	Yes
ENSMUST00000021271	Per1	−0.67208	0.00005	0.00141	0.1138	Yes
ENSMUST00000063446	Sphk1	−0.50306	0.09151	0.51061	0.1803	Yes

## Data Availability

The data used to support the findings of this study are available from the corresponding author upon request.

## Abbreviations

ACR: Albumin creatinine ratio
AGEs: Advanced glycation products
DEGs: Differentially expressed genes
DN: Diabetic nephropathy
ESRD: End-stage renal disease
GBM: Globular basement membrane
GSEA: Gene set enrichment analysis
GFR: Glomerular filtration rate
GO: Gene Ontology
KEGG: Kyoto Encyclopedia of Genes and Genomes
lncRNAs: Long noncoding RNAs
PKC: Protein kinase C
PPAR*γ*: Peroxisome proliferator-activated receptor gamma
RAAS: Renin-angiotensin-aldosterone system
ROS: Reactive oxygen species
TGF-*β*: Transforming growth factor-beta
TZDs: Thiazolidinediones.

## Supplementary Materials

Supplementary Materials Supplementary Table 1: significantly upregulated mRNAs in dbdb mice compared to the control group. Supplementary Table 2: significantly downregulated mRNAs in dbdb mice compared to the control group. Supplementary Table 3: significantly upregulated mRNAs in dbR mice compared to the dbdb group. Supplementary Table 4: significantly downregulated mRNAs in dbR mice compared to the dbdb group. Supplementary Table 5: mRNAs significantly upregulated by diabetic nephropathy yet reversed following rosiglitazone treatment. Supplementary Table 6: mRNAs significantly downregulated by diabetic nephropathy yet reversed following rosiglitazone treatment. Supplementary Table 7: significantly upregulated lncRNAs in dbdb mice compared to the control group. Supplementary Table 8: significantly downregulated lncRNAs in dbdb mice compared to the control group. Supplementary Table 9: significantly upregulated lncRNAs in dbR mice compared to the dbdb group. Supplementary Table 10: significantly downregulated lncRNAs in dbR mice compared to the dbdb group. Supplementary Table 11: lncRNAs significantly upregulated by diabetic nephropathy yet reversed following rosiglitazone treatment. Supplementary Table 12: lncRNAs significantly downregulated by diabetic nephropathy yet reversed following rosiglitazone treatment. Supplementary Table 13: differentially expressed lncRNAs colocated with genes. Supplementary Table 14: differentially expressed lncRNAs coexpressed with genes. Supplementary Table 15: top 20 significantly enriched GO terms associated with differentially expressed mRNAs in the kidneys of dbdb mice compared to the controls. Supplementary Table 16: top 20 significantly enriched GO terms associated with differentially expressed mRNAs in the kidneys of dbR mice compared to the dbdb group. Supplementary Table 17: top 20 significantly enriched GO terms associated with differentially expressed lncRNAs coexpressed with genes in the kidneys of dbdb mice compared to the controls. Supplementary Table 18: top 20 significantly enriched GO terms associated with differentially expressed lncRNAs coexpressed with genes in the kidneys of dbR mice compared to the dbdb group. Supplementary Table 19: top 20 enriched KEGG pathways associated with differentially expressed mRNAs in dbdb mice compared to the controls. Supplementary Table 20: top 20 enriched KEGG pathways associated with differentially expressed mRNAs in dbR mice compared to dbdb mice. Supplementary Table 21: top 20 enriched KEGG pathways associated with differentially expressed lncRNAs colocated with genes in dbdb mice compared to controls. Supplementary Table 22: top 20 enriched KEGG pathways associated with differentially expressed lncRNAs colocated with genes in dbR mice compared to dbdb mice. Supplementary Table 23: top 20 enriched KEGG pathways associated with differentially expressed lncRNAs coexpressed with genes in dbdb mice compared to the controls. Supplementary Table 24: top 20 enriched KEGG pathways associated with differentially expressed lncRNAs coexpressed with genes in dbR mice compared to dbdb mice. Supplementary Table 25: gene sets significantly upregulated in dbdb mice compared to controls as determined by GSEA. Supplementary Table 26: gene sets significantly upregulated in controls compared to dbdb mice as determined by GSEA. Supplementary Table 27: gene sets significantly upregulated in dbR mice compared to dbR mice as determined by GSEA. Supplementary Table 28: gene sets significantly upregulated in dbdb mice as compared to dbR mice as determined by GSEA. Supplementary Table 29: primer sequences used in the qRT-PCR analysis to validate lncRNA expression. Supplementary Table 30: locations and sequences of differentially expressed novel lncRNAs identified via RNA-seq.
